# Anaemia and red blood cell transfusion in women with placenta accreta spectrum: an analysis of 38,060 cases

**DOI:** 10.1038/s41598-024-55531-6

**Published:** 2024-02-29

**Authors:** Jan Andreas Kloka, Benjamin Friedrichson, Thomas Jasny, Lea Valeska Blum, Suma Choorapoikayil, Oliver Old, Kai Zacharowski, Vanessa Neef

**Affiliations:** Department of Anaesthesiology, Intensive Care Medicine and Pain Therapy, Goethe University Frankfurt, University Hospital, Theodor-Stern Kai 7, 60590 Frankfurt, Germany

**Keywords:** Medical research, Risk factors

## Abstract

Placenta accreta spectrum (PAS) has become a significant life-threatening issue due to its increased incidence and associated morbidity and mortality. Pregnancy is often associated with states of anaemia, and severe maternal haemorrhage represents a major risk factor for red blood cell (RBC) transfusion. The present study retrospectively analyzed the prevalence of anaemia, transfusion requirements and outcome in women with PAS. Using data from the German Statistical Office pregnant patients with deliveries hospitalized between January 2012 and December 2021 were included. Primary outcome was the prevalence of anemia and administration of RBCs. Secondary outcome were complications in women with PAS who received RBC transfusion. In total 6,493,606 pregnant women were analyzed, of which 38,060 (0.59%) were diagnosed with PAS. The rate of anaemia during pregnancy (60.36 vs. 23.25%; p < 0.0001), postpartum haemorrhage (47.08 vs. 4.41%; p < 0.0001) and RBC transfusion rate (14.68% vs. 0.72%; p < 0.0001) were higher in women with PAS compared to women without PAS. Women with PAS who had bleeding and transfusion experienced significantly more peripartum complications than those who did not. A multiple logistic regression revealed that the probability for RBC transfusion in all pregnant women was positively associated with anaemia (OR 21.96 (95% CI 21.36–22.58)). In women with PAS, RBC transfusion was positively associated with the presence of renal failure (OR 11.27 (95% CI 9.35–13.57)) and congestive heart failure (OR 6.02 (95% CI (5.2–7.07)). Early anaemia management prior to delivery as well as blood conservation strategies are crucial in women diagnosed with PAS.

## Introduction

Placenta accreta spectrum (PAS) is defined as a morbidly adherent placenta and includes placenta accreta, placenta increta, and placenta percreta. The incidence of PAS has been gradually increasing and is currently estimated to be present in one of every 533 births (0.18%) in the United States, mainly due to an increasing rate of caesarean sections^1^. In a recent meta-analysis including 29 articles across countries overall prevalence ranged from 0.01 to 1.10% with an overall pooled prevalence of 0.17% (95% confidence interval (CI) 0.14–0.19)^2^. A German study reports a PAS prevalence of 2.49 out of 1000 births (0.25%) in Germany^3^.

Abnormal placentation is one of the most common causes of major haemorrhage and therefore a major risk factor for increased maternal morbidity and mortality^4^. A study by Wright et al. on transfusion requirements in women diagnosed with PAS showed that median blood loss during delivery is 3000 ml^5^. A recent meta-analysis by Miller et al. in 2023 included 20 studies with 1,091 cases of PAS and revealed a weighted mean number of red blood cell (RBC) transfusions of 5.19 (95% CI 4.12–6.26) per patient^6^. Among unscheduled, emergent deliveries in women diagnosed with PAS transfusion requirements are even higher (median (25;75 IQR) 8.0 (3.0; 10.0) units) with a transfusion rate of 92.6%^7^. The increased morbidity in women diagnosed with PAS and maternal haemorrhage are often aggravated by associated coagulopathies and anaemia during pregnancy. About 30.0% of women with PAS undergoing hysterectomy develop coagulopathy, associated with changes in platelet count, international normalized ratio, fibrinogen levels and increased RBC transfusion requirements^8,9^.

Anaemia during pregnancy is a frequent, serious global health problem and affects about 42.0% of all pregnant women worldwide^10^. Anaemia during pregnancy is associated with worse peripartum outcomes, increased transfusion requirements and postpartum depression or fatigue^11^. Anemia can be caused by increased iron demands, placental abruption and peripartum blood losses^12^.

Patients with PAS and antepartum bleedings are at higher risk of baseline anaemia and increased transfusion requirements. Detection and treatment of anaemia in obstetrics are strongly recommended in order to reduce severe anaemia, the risk for RBC transfusion and decrease the patient´s morbidity and mortality^13^. With the intention to improve the management of women diagnosed with PAS, the present study aims to examine anaemia rate, RBC transfusion and outcomes in women diagnosed with PAS over the last 10 years in Germany, using a large database.

## Material and methods

### Inclusion criteria

All hospitalized women for delivery between January 1st 2012 and December 31st 2021 in Germany were included in the study.

### Exclusion criteria

The code O43.9 (pathological condition of the placenta, unspecified) was excluded from the study, as it serves as a collective code for any pathological condition of the placenta and therefore no relation to PAS can be drawn.

### Data availability

Hospitals in Germany are required by law to report diagnoses according to the International Statistical Classification of Diseases and Related Health Problems (ICD) codes and the International Statistical Classification of Procedures (OPS) codes^14^. According to §21 of the German Hospital Finance Law (KHG), all German hospitals are required to submit this data in anonymous form to the Institute for Hospital Remuneration (InEK) for further development of the DRG system. InEK further transfers the data to the Federal Statistical Office, where the data is made available for researchers with a signed contract for data usage. As the reporting of ICD-10 and OPS codes is mandatory by law and reimbursement is based on this data, there is a strong incentive to capture all births in German hospitals. The German Federal Statistical Office stores the data on its local site. Individual patient and hospital identifiers were not available to the authors.

### Definitions and data acquisition

All age groups and data from 2012 to 2021 were included. More recent data were not available due to accounting aspects and the internal data validation processes of the Federal Statistical Office. The Federal Statistical Office processes the data, assesses their validity, and releases them for further scientific analysis.

Data collected included demographics (e.g., age, gestational age, mode of delivery, anticoagulation therapy), comorbidities (e.g., essential hypertension, gestational hypertension, diabetes during pregnancy, obesity, different forms of anaemia), complications (e.g., postpartum renal failure, pneumonia, death, miscarriage, hospital length of stay (LOS), cardiopulmonary resuscitation), and obstetric problems (e.g., prepartum,- intrapartum,- and postpartum haemorrhage (PPH), anaemia due to acute bleeding).

In Germany, PPH is defined as blood loss > 500 ml after vaginal delivery and > 1000 ml after caesarean section^15^. Diagnoses were coded according to the 10th revision of the International Classification of Diseases and procedures according to the International Classification of Procedures in Medicine (version 2020). The assignment of data collected as well as OPS and ICD-10 codes to the corresponding procedures and diseases can be found in Supplemental Table 1.

### Definition of anaemia

In Germany, anemia during pregnancy is defined as Hb value < 11 g/dl in the first and third trimester, and Hb value < 10.5 g/dl in the second trimester^16^.

There are different forms of coding of anaemia in ICD-10. Coding of anaemia can be divided into general codes for anaemia and specific codes for anaemia during pregnancy (marked with an “O” for obstetrics, see Supplemental Table 1). As the general forms of anaemia were used during the coded case of delivery, anaemia was ensured to be present during pregnancy.

### Statistical analysis

Categorical variables are expressed as absolute numbers and percentages. Continuous variables were tested for normality. All continuous variables (age, LOS, and mechanical ventilation) were non-normally distributed. Hence, continuous variables were presented as the median with 25% and 75% quartiles (Q1;Q3). Group differences between categorical variables were tested for statistical significance with the chi-square test, and for continuous variables with Wilcoxon-rank-sum test. Groups for predefined comorbidities, birth mode, anemia, bleeding and complications were the RBC transfusion group and the non-RBC transfusion group and using their respective ICD and OPS codes as defined in Supplemental Table 1. Two separate multiple logistic regression models were conducted. In the first model, the probability for RBC transfusion in all pregnant women was estimated while controlling for age, gestational hypertension, renal failure, congestive heart failure, PAS, Caesarean section, and anaemia during pregnancy. In the second model, the probability for RBC transfusion within the PAS population was analyzed, adjusting for age, gestational hypertension, congestive heart failure, Caesarean section, and anaemia during pregnancy. The statistical significance level was set to 5%. Excel 2019 (Microsoft Corp., Seattle, WA, USA) was used for data handling and SAS (Version 9.4M6, SAS Institute Inc., Cary, NC, USA) for statistical analysis.

### Ethics approval and consent to participate

Due to institutional anonymization, no conclusions can be drawn about individual patients and therefore the General Data Protection Regulation does not apply. According to §21KHEntgG, anonymized reimbursement data are free for scientific use. The Ethics Committee of the University Hospital Frankfurt waived the requirement for approval for this study (Ref: 2022-766). All data processing was performed in accordance with the tenets of the Declaration of Helsinki.

## Results

In total 6,493,606 pregnant women were hospitalized between January 1st 2012 and December 31st 2021. Of these, data from 38,060 women with diagnosed PAS were analyzed in this study.

### Demographic characteristics of all pregnant patients

Median (25;75 IQR) age in all women was 31 (27;34) years. In case of bleeding, obstetric haemorrhage occurred mostly in the postpartum period (4.66%) compared to intrapartum haemorrhage (0.53%) and prepartum haemorrhage (0.39%). “Anaemia during pregnancy” was diagnosed in 23.47%, “any other form of anaemia” in 11.93%, and “anaemia due to acute bleeding situations” in 10.38% of all women. Further patient characteristics are depicted in Table 1.

**Table 1:** Demographic characteristics.

	Obstetric patients	
Total patients, n	6,493,606	
Age, years; median [Q1;Q3]	31 [27;34]	
Age groups		
10–14; n, %	1 155	0.02
15–19; n, %	130 114	2.00
20–24; n, %	694 795	10.70
25–29; n, %	1 770 314	27.26
30–34; n, %	2 330 564	35.89
35–39; n, %	1 285 187	19.79
40–44; n, %	265 836	4.09
45–49; n, %	13 729	0.21
50–54; n, %	876	0.01
55–59; n, %	96	0.00
60–64; n, %	5	0.00
Comorbidities		
PAS; n, %	38 060	0.59
Essential hypertension; n, %	11 546	0.18
Gestational hypertension; n, %	76 343	1.18
Diabetes during pregnancy; n, %	399 493	6.15
Obesity; n, %	161 215	2.48
Grade I; n, %	26 704	0.41
Grade II; n, %	23 269	0.36
Grade III; n, %	26 829	0.41
Anaemia		
Vitamine B12-, folic acid-, any other dietary anaemia; n, %	1 216	0.02
Any other form of anaemia; n, %	774 640	11.93
Anaemia due to acute bleeding; n, %	673 802	10.38
Anaemia during pregnancy; n, %	1 524 171	23.47
Anticoagulation therapy; n, %	9 301	0.14
Bleeding		
Prepartum haemorrhage; n, %	25 422	0.39
Intrapartum haemorrhage; n, %	27 926	0.43
Postpartum haemorrhage; n, %	302 737	4.66

*PAS* Placenta accreta spectrum.

All variables are expressed as n (%) or Median [Q1;Q3].

### Comparison of the PAS and non-PAS group

Median (25;75 IQR) age in all women diagnosed with PAS was 32 (29;36) years. Comorbidities such as gestational hypertension (1.42 vs. 1.17%; p < 0.0001) and pre-existing diabetes during pregnancy (6.76 vs. 6.15%; p < 0.0001) were slightly more frequent in the PAS group compared to the non-PAS group. The rate of “anaemia during pregnancy” was significantly higher in women diagnosed with PAS compared to women without PAS (60.36 vs. 23.25%; p < 0.0001). In addition, the rate of “any other form of anaemia” (45.98 vs. 11.73%; p < 0.0001) and “anaemia due to acute bleeding” (43.99 vs. 10.18%; p < 0.0001) was diagnosed more often in the PAS group compared to the non-PAS group. Significant differences were found in the occurrence of PPH between the PAS and non-PAS group (47.08 vs. 4.41%; p < 0.0001).

Last, median hospital LOS was significantly longer in women with PAS compared to women without PAS (4.21 [3.24; 5.8] vs. 3.53 [3.51; 4.68] days; p < 0.0001) (Table 2).

**Table 2:** Comparison of the PAS and non-PAS group.

	PAS		Non-PAS		p-value
Total patients; n, %	38,060	0.59	6,455,546	99.41	
Age, years; median [Q1;Q3]	32 [29;36]		31 [27;34]		< 0.0001
Gestational age [weeks]					
< 5; n, %	*	*	*	*	
15–13; n, %	*	*	*	*	
14–19; n, %	74	6.03	1228	94.32	
20–25; n, %	770	3.24	23,743	96.86	
26–33; n, %	2376	1.92	123,464	98.11	
34–36; n, %	3462	1.3	265,439	98.71	
37–41; n, %	27,686	0.51	5,417,061	99.49	
> 41; n, %	3665	0.61	604,765	99.4	
Unspecified; n, %	31	0.52	6000	99.49	
Birth mode					
Vaginal delivery; n, %	10,571	27.77	4,378,387	67.82	< 0.0001
Caesarean section; n, %	28,498	74.88	2,276,208	35.26	< 0.0001
Comorbidities					
Essential hypertension; n, %	108	0.28	11,438	0.18	< .0001
Gestational hypertension; n, %	541	1.42	75,802	1.17	< .0001
Diabetes during pregnancy; n, %	2573	6.76	396,920	6.15	< .0001
Obesity; n, %	909	2.39	160,306	2.48	0.2355
Grade I; n, %	199	0.52	26,505	0.41	0.0006
Grade II; n, %	143	0.38	23,126	0.36	0.5692
Grade III; n, %	136	0.36	26,693	0.41	0.0886
Anaemia					
Vitamine B12-, folic acid-, any other dietary anaemia; n, %	12	0.03	1204	0.02	0.0671
Any other form of anaemia; n, %	17,500	45.98	757,140	11.73	< 0.0001
Anaemia due to acute bleeding; n, %	16,741	43.99	657,061	10.18	< 0.0001
Anaemia during pregnancy; n, %	22,972	60.36	1,501,199	23.25	< 0.0001
Anticoagulation therapy; n, %	94	0.25	9207	0.14	< 0.0001
Bleeding					
Prepartum haemorrhage; n, %	344	0.90	25,078	0.39	< 0.0001
Intrapartum haemorrhage; n, %	784	2.06	27,142	0.42	< 0.0001
Postpartum haemorrhage; n, %	17,919	47.08	284,818	4.41	< 0.0001
Blood products					
RBCs; n, %	5588	14.68	46,206	0.72	< 0.0001
Fresh frozen plasma; n, %	1 447	3.80	7041	0.11	< 0.0001
Platelets; n, %	364	0.96	3203	0.05	< 0.0001
Prothrombin complex concentrate; n, %	478	1.26	2742	0.04	< 0.0001
Fibrinogen; n, %	2271	5.97	13,216	0.20	< 0.0001
Massive blood transfusion; n, %	293	0.77	1137	0.02	< 0.0001
Complications					
Pneumonia; n, %	45	0.12	2417	0.04	< 0.0001
Postpartum renal failure; n, %	78	0.20	2665	0.04	< 0.0001
Death; n, %	14	0.04	249	0.00	< 0.0001
Miscarriage (child born dead); n, %	493	1.30	24,612	0.38	< 0.0001
Length of hospital stay, days; median [Q1;Q3]	4.21 [3.24;5.8]		3.53 [3.52;4.68]		< 0.0001
Cardiopulmonary resuscitation; n, %	40	0.11	605	0.01	< 0.0001

*PAS* Placenta accreta spectrum, *RBC* Red blood cell.

*Censored ≤ 3 patients.

All variables are expressed as n (%) or Median [Q1;Q3].

### Administration of blood products

Overall, blood products were administered more frequently in women diagnosed with PAS compared to women without PAS. Allogeneic RBCs were transfused significantly more often in the PAS group compared to the non-PAS group (14.68 vs. 0.72%; p < 0.0001). In addition, massive blood transfusion (defined as > 5 RBCs) was conducted more frequently in women with PAS compared to women without PAS (0.77 vs. 0.02%; p < 0.0001) (Table 2).

### Comparison of RBC transfusion and non-RBC transfusion in women diagnosed with PAS

The rate of “anaemia during pregnancy” (90.43 vs. 55.18%; p < 0.0001), “any other form of anaemia” (92.34 vs. 38.0%; p < 0.0001) and “anaemia due to acute bleeding” (91.93 vs. 35.74%; p < 0.0001) were significantly higher in women with PAS who received RBC transfusion than those who did not. Complications such as pneumonia (0.57 vs. 0.04%; p < 0.0001) and renal failure (1.04 vs. 0.06%; p < 0.0001) occurred significantly more often in transfused women with PAS compared to those who did not. Also, median hospital LOS was significantly longer in women with PAS who received RBC transfusion compared to those who women without RBC transfusion (6.08 [4.36;9.27]] vs 4.07 [3.17;5.28] days; p < 0.0001) (Table 3).

**Table 3:** Comparison of RBC transfusion and non-RBC transfusion in women with PAS.

	Non-RBC		RBC		p-value
Total patients; n, %	32,472	85.31	5588	14.68	
Age, years; median [Q1;Q3]	32 [29;36]		33 [29;36]		< 0.0001
Comorbidities					
Essential hypertension; n, %	75	0.23	33	0.59	< 0.0001
Gestational hypertension; n, %	456	1.40	85	1.52	< 0.0001
Diabetes during pregnancy; n, %	1969	6.06	428	7.66	< 0.0001
Obesity; n, %	769	2.37	140	2.51	0.5350
Grade I; n, %	169	0.52	30	0.54	0.8751
Grade II; n, %	121	0.37	22	0.39	0.8120
Grade III; n, %	111	0.34	25	0.45	0.2219
Anaemia					
Vitamine B12-, folic acid-, any other dietary anaemia; n, %	6	0.02	6	0.11	0.0005
Any other form of anaemia; n, %	12 340	38.00	5 160	92.34	< 0.0001
Anaemia due to acute bleeding; n, %	11 604	35.74	5 137	91.93	< 0.0001
Anaemia during pregnancy; n, %	17 919	55.18	5 053	90.43	< 0.0001
Anticoagulation therapy; n, %	68	0.21	26	0.47	0.0004
Bleeding					
Prepartum haemorrhage; n, %	257	0.79	87	1.56	< .0001
Intrapartum haemorrhage; n, %	401	1.23	383	6.85	< .0001
Postpartum haemorrhage; n, %	14 021	43.18	3 898	69.76	< .0001
Complications					
Pneumonia; n, %	13	0.04	32	0.57	< .0001
Postpartum renal failure; n, %	20	0.06	58	1.04	< .0001
Death; n, %	*	*	*	*	< .0001
Miscarriage (child born dead); n, %	424	1.31	69	1.23	0.6648
Length of stay, days; median [Q1;Q3]	4.07 [3.17;5.28]		6.08 [4.36;9.27]		< .0001
Cardiopulmonary resuscitation; n, %	6	0.02	34	0.61	< .0001

*PAS* Placenta accreta spectrum, *RBC* Red blood cell.

*Censored ≤ 3 patients.

All variables are expressed as n (%) or Median [Q1;Q3].

Multiple logistic regression showed that the probability of RBC transfusion in pregnant women was positively associated with PAS with an odds ratio (OR) of 11.93 (95% CI 11.55–12.32). “Anaemia during pregnancy” had an OR of 21.96 (95% CI 21.36–22.58), and renal failure had an OR of 11.63 (95% CI 9.61–14.07). The probability of RBC transfusion in women with PAS was positively associated with “anaemia during pregnancy” with an OR of 23.1 (95% CI 22.5–23.72) renal failure with an OR of 11.27 (95% CI 9.35–13.57) and congestive heart failure with an OR of 6.02 (95% CI 5.2–7.07) (Table 4).

**Table 4:** Logistic regression models of blood product utilization among all obstetric patients (A) and women with PAS (B).

RBC administration	OR (95% CI)
(A) In obstetrics	
Age	1.01 (1.01–1.01)
Gestational hypertension	2.79 (2.49–3.13)
Renal failure	11.63 (9.61–14.07)
Congestive heart failure	13.17 (11.32–15.33)
PAS	11.93 (11.55–12.32)
Caesarean section	1.47 (1.44–1.49)
Anaemia during pregnancy	21.96 (21.36–22.58)
(B) In PAS	
Age	1.01 (1.00–1.02)
Gestational hypertension	2.64 (2.37–2.95)
Renal failure	11.27 (9.35–13.57)
Congestive heart failure	6.02 (5.2–7.07)
Caesarean section	1.32 (1.3–1.34)
Anaemia during pregnancy	23.1 (22.5–23.72)

*OR* Odds ratio, *CI* Confidence interval, *PAS* Placenta accreta spectrum, *RBC* Red blood cell.

### Distribution of rate of PAS, anaemia, PPH, bleeding and hysterectomy by hospital size based on the number of deliveries per year

When considering the size of the hospital (defined by the number of deliveries per year), most women with PAS delivered in a hospital with > 3501 births per year (1.08%). In the hospitals with 3501–4,000 deliveries, the rate of “anaemia during pregnancy” (57.70%) and hypovolaemic shock (1.11%) were one of the lowest, compared to the other hospitals. The rate of PPH (56.47%) and hysterectomy (3.88%) was the highest in hospitals with 3001–3500 deliveries per year. All variables based on the hospital size are depicted in Table 5.

**Table 5:** Distribution of hospitals by number of deliveries with rate of PAS, anaemia, postpartum haemorrhage, bleeding and hysterectomy.

	Deliveries	PAS		Anaemia during pregnancy		PPH		Hypovolaemic shock		Hysterectomy	
	n	n	%	n	%	n	%	n	%	n	%
Number of deliveries											
≤ 500	643,252	2954	0.46	1732	58.63	1087	36.8	42	1.42	45	1.52
501–1000	1,798,144	9199	0.51	5564	60.48	3980	43.27	125	1.36	193	2.1
1001–1500	1,482,611	8321	0.56	5294	63.62	4019	48.3	122	1.5	198	2.38
1501–2000	1,094,689	6975	0.64	4350	62.37	3399	48.73	127	1.82	226	3.24
2001–2500	629,856	4307	0.68	2466	57.26	2283	53.01	57	1.32	140	3.25
2501–3000	398,655	2630	0.66	1321	50.23	1431	54.41	40	1.52	82	3.12
3001–3500	194,391	1236	0.64	767	62.06	698	56.47	9	0.73	48	3.88
3501–4000	92,098	993	1.08	573	57.7	511	51.46	11	1.11	33	3.32
4001–4500	*	*	*	*	*	*	*	*	*	*	*
> 4500	*	*	*	*	*	*	*	*	*	*	*

Table 5 depicts the rate of PAS, anaemia, PPH, hypovolaemic shock and hysterectomy by hospital size based on the number of deliveries. Deliveries are calculated over a period of 10 years (2012–2021).

*PAS* Placenta accreta spectrum, *PPH* Postpartum haemorrhage.

*Censored ≤ 3 patients.

## Discussion

This retrospective study includes a cohort of 6,493,606 pregnant women hospitalized between January 2012 and December 2021 in Germany. Of these, 38,606 women (0.59%) were diagnosed with PAS. In women with PAS the rate of PPH, “anaemia during pregnancy” and RBC transfusion were significantly higher compared to women without PAS. Transfusion of RBCs in patients with PAS was associated with increased single complications in the peripartum period. Multiple logistic regression revealed that the probability of RBC transfusion in women with PAS was positively associated with presence of anaemia, renal failure and congestive heart failure.

In Germany, management of PAS approach depends on the extent of placental invasion. In case of extensive placental invasion, mostly caesarean hysterectomy is conducted. In case of a partial affected area an uterus-preserving approach with partial excision of the affected area is recommended. If fertility preservation is desired, or there is a high risk of surgical complications, it is also possible to leave the placenta in situ and consider a subsequent embolization of the uterine arteries^15^.

Irrespective of the surgical approach the presence of PAS is often accompanied with severe haemorrhage^4^. Our findings of an increased rate of PPH in women with PAS compared to women without PAS (47.08 vs. 4.41%; p < 0.0001) are in line with recently published studies. A study by Bailit et al. reveals that 33.0% of all women with PAS experience increased blood losses^17^. Another study by Esakoff et al. demonstrates, that women with placenta accreta had an OR of 89.6 (95% CI 19.44–412.95) for estimated blood loss > 2000 ml, and an OR of 29.63 (95% CI 8.20–107.00) for RBC transfusion^18^. In emergency situations intraoperative blood loss may also exceed 8,000 ml in women with PAS^19^. In addition, antepartum bleeding occurred significantly more often in women with PAS compared to women without PAS (0.9 vs. 0.39%; p < 0.0001). Bleeding before delivery might be explained e.g. by coexisting placenta previa or urinary bladder invasion of the placenta^20^.

Recent studies demonstrate that childbirth in women with PAS is associated with increased need for RBC transfusion (54.0–95.5%)^3,21^. In a retrospective audit of women diagnosed with PAS, a median [range] number of four RBC units [0–10] were transfused^22^. There is evidence, that RBC transfusion is associated with increased postoperative complications e.g. longer hospital length of stay^23^. In our study, the rate of RBC transfusion (14.68 vs. 0.72; p < 0.0001) and massive blood transfusion (0.77 vs. 0.02%; p < 0.0001) were also higher in women with PAS compare to women without PAS). The majority of all transfused women with PAS received 1–5 RBC units (84.43%). In addition, 10.43% received 6–10, 3.17% 11–15, and 1.36% 16–23 RBC units (Supplemental Table 2). Our results show, that the probability of RBC transfusion in women with PAS was positively associated with presence of “anaemia during pregnancy” (OR 23.1 (95% CI 22.5–23.72)), renal failure (OR 11.27 (95% CI 9.35–13.57)) and congestive heart failure (OR 6.02 (95% CI 5.2–7.07)). Cardiac failure may be associated with the need for cardiac assist devices and anticoagulation therapy. Also, renal failure may lead to impaired production of erythropoietin and anaemia. These reasons may also increase the risk of RBC transfusion.

When considering the number of deliveries of the hospital our results show, that PPH is the highest in larger hospitals with 2,001–3,500 births per year (53.01–56.47%). Interestingly, despite increased rates of PPH, the rate of hypovolaemic shock is the lowest in larger hospitals with 2001–2500 (1.32%) and 3001–4000 (0.73–1.11%) births per year. Increased rates of PPH might be explained by the treatment of more complex PAS cases in bigger and specialized hospitals. A recently published study in the use of cell salvage during peripartum haemorrhage revealed, that cell salvage is used more frequently in hospitals with > 1337 births per year^24^. Especially the use of cell salvage in women with anticipated high risk for increased blood losses is highly important^13^, along with other measures such as use of tranexamic acid^25^. During caesarean hysterectomy large amounts of blood losses may occur^17^. Our result show, that in hospitals with a high rate of hysterectomies (3.88%) and 3001–3500 deliveries per year, the rate of hypovolaemic shock is the lowest (0.73%). These findings may be explained by the center’s surgical experience as well as the use of blood conservation strategies (e.g. cell salvage).

Regarding anaemia prevalence our study demonstrates, that the rate of “anaemia during pregnancy” was one of the lowest in large hospitals with 3501–4000 births per year (57.70%). In small hospitals with 1001–1500 births per year it was the highest (63.62%). Various studies have demonstrated that anaemia during pregnancy is associated with increased morbidity and mortality^26–28^. In our study, “anaemia during pregnancy” was significantly higher in the PAS group compared to the non-PAS group (60.36 vs. 23.25%; p < 0.001) as well as in transfused women with PAS compared to non-transfused women with PAS (90.43 vs. 55.18%; p < 0.0001). Since women with PAS often suffer from insufficient iron stores (e.g. peripartum bleeding), iron management during pregnancy is crucial^13^. In general, national and international guidelines in terms of Patient Blood Management (PBM) recommend screening for anaemia in the antepartum period as well as at any time during pregnancy if anaemia is present^13,29^. The clinical guideline of the American College of Obstetrics and Gynaecologists (ACOG) especially on women with PAS emphasizes the importance of maximization of antepartum haemoglobin values and the evaluation for management of anaemia during pregnancy^30^. As prepartum diagnosis of (iron deficiency) anaemia requires further measurements of iron parameters^31^ and intrapartum anaemia may be avoided by the use of blood conservation strategies, these two reasons might account for an increased rate of “anaemia during pregnancy” in smaller hospitals compared to larger hospitals with more resources available.

Our analyses reveal that RBC transfusion in bleeding women with PAS is associated with increased single complications. The rates of pneumonia (0.57 vs. 0.04%; p < 0.0001), renal failure (1.04 vs. 0.06%; p < 0.0001) and hospital LOS (4.21 [3.24; 5.8] vs. 3.53 [3.51; 4.68] days; p < 0.0001) were higher in transfused women with PAS compared to non-transfused women with PAS. In addition, there are morbidities directly associated with transfusion reactions (e.g. ABO/rhesus incompatibility). In general, these transfusion reactions are rare in all transfused pregnant women (0.03%). Our analysis reveals, that women with transfusion reactions suffered significantly more often from renal failure (12.9 vs. 1.43%; p < 0.0001), dialysis (12.9 vs. 1.18%; p < 0.0001, cardiopulmonary resuscitation (9.68 vs. 0.68%; p < 0.0001) or the need for mechanical ventilation (25.81 vs. 10.56%; p = 0.006) compared to transfused women without transfusion reaction (Supplemental Table 3).

To summarized, the implementation of PBM in obstetrics has proved to reduce anaemia, the need for RBC transfusion and decrease their associated risks and complications^13,32,33^.

## Limitations

To our knowledge, this study is the largest survey of pregnant women over a period of 10 years in Europe. One of the limitations inherent in the present study pertains to the retrospective nature and utilization of secondary reimbursement data. Reimbursement data have a correlation with the medical cases in the hospital^34^. However, it cannot be entirely precluded that certain conditions or events might be either over- or under-represented due to reimbursement considerations. Nonetheless, there exists an increased incentive for accurate documentation, as the medical service of the health insurance funds conducts audits on hospital reimbursements. The parameters chosen for this study were based on their high medical relevance, aiming to minimize the occurrence of coding errors. Due to the large sample size, possibly misreported data should be largely counterbalanced. It is noteworthy to mention, that the exact time point of blood product administration and coding of anaemia is not available with this data (e.g. before or after delivery). However, blood transfusion occurred during the hospital stay of delivery. Also, blood loss during vaginal delivery, caesarean section or hysterectomy cannot be coded and thus, is not available. As the amount of bleeding may give further important information on transfusion requirements, this variable should be acknowledged in future studies. Furthermore, transfusion may be directly associated with specific complications such as transfusion-related acute lung injury (TRALI) or transfusion-associated circulatory overload (TACO). As these morbidities are not coded separately, this data is not available. However, these specific transfusion related complications should be considered in future studies. Data were collected in a structured and representative manner according to the Declaration of Helsinki. Laboratory findings or medication are not coded for reimbursement and are therefore not available for analysis. Finally, data only provide information on in-hospital pregnancies and deliveries.

## Conclusions

The prevalence of anaemia during pregnancy in women with PAS is high (60.36%). In addition, the rate of PPH, RBC transfusion and administration of blood products are higher in women with PAS compared to women without PAS. Transfused women with PAS have an increased rate of peripartum complications compared to non-transfused women with PAS. Multiple logistic regression revealed that the probability for RBC transfusion in all pregnant women is positively associated with anaemia. In women with PAS RBC transfusion is positively associated with the presence of anaemia, renal failure and congestive heart failure. Therefore, it is of high relevance especially in women with PAS, to identify and subsequently treat prepartum anaemia early prior to delivery.

### Supplementary Information

Supplementary Table 1.

Supplementary Table 2.

Supplementary Table 3.

## Data Availability

The data on which the results of this study are based are available from the Federal Statistical Office with the restrictions applied. The dataset was used under licence for the current study and is therefore not generally accessible. However, the data are available from the corresponding author on reasonable request and with permission from the Federal Statistical Office.
