# Supplementary material for: Anaemia and red blood cell transfusion in women with placenta accreta spectrum: an analysis of 38,060 cases
Source: Sci Rep. 2024 Feb 29;14:4999. doi: 10.1038/s41598-024-55531-6 (PMC10904858; doi:10.1038/s41598-024-55531-6)
Supplement: Supplementary file 3 — Supplementary Table 3. [file 41598_2024_55531_MOESM3_ESM.docx]

| Pregnant women with RBC transfusion from 2010-2022 and AB0/Rh incompatibility | | | |  |  |
| --- | --- | --- | --- | --- | --- |
|  | no transfusion incompatibility | | transfusion incompatibility | | p-value |
|  |  |  |  |  |  |
| Total patients; n,% | 100.729 | 99,97 | 31 | 0,03 |  |
| Age, years [Q1;Q3] | 31 [27;35] | | 30 [26;34] | | 0,4351 |
| AB0 incompatibility; n,% |  |  | 27 | 87,1 |  |
| Rh incompatibility; n,% |  |  | 4 | 12,9 |  |
|  |  |  |  |  |  |
| **Gestational age [weeks]** |  |  |  |  |  |
| 14 - 19; n,% | * | * | * | * |  |
| 20 - 25; n,% | * | * | * | * |  |
| 26 - 33; n,% | 9.553 | 9,48 | 6 | 19,35 |  |
| 34 - 36; n,% | 8.394 | 8,33 | 0 | 0 |  |
| 37 - 41; n,% | 54.866 | 54,47 | 16 | 51,61 |  |
| > 41; n,% | * | * | * | * |  |
| unspecified | * | * | * | * |  |
|  |  |  |  |  |  |
| **Anaemia** |  |  |  |  |  |
| Vitamine B12-, folic acid-, any other dietary anaemia; n,% | 10.666 | 10,59 | 8 | 25,81 | 0,67 |
| Any other form of anaemia; n,% | 84.697 | 84,08 | 28 | 90,32 | 0,34 |
| Anaemia due to acute bleeding; n,% | 81.032 | 80,45 | 25 | 80,65 | 0,92 |
| Anaemia during pregnancy; n,% | 78.694 | 78,12 | 25 | 80,65 | 0,73 |
| Anticoagulation therapy; n,% | 487 | 0,48 | 0 | 0 |  |
|  |  |  |  |  |  |
| **Bleeding** |  |  |  |  |  |
| Praepartum haemorrhage; n,% | 1.543 | 1,53 | 0 | 0 |  |
| Intrapartum haemorrhage; n,% | 3.410 | 3,39 | 3 | 9,68 | 0,05 |
| Postpartum haemorrhage; n,% | 44.021 | 43,7 | 11 | 35,48 | 0,36 |
|  |  |  |  |  |  |
| **Number of RBCs** |  |  |  |  |  |
| 1 – 5; n,% | 92.101 | 91,43 | 24 | 77,42 |  |
| 6 – 10; n,% | 6.094 | 6,05 | 5 | 16,13 |  |
| 11 – 15; n,% | 1.393 | 1,38 | 0 | 0 |  |
| 16 – 23; n,% | * | * | * | * |  |
| 24 – 31; n,% | 217 | 0,22 | 0 | 0 |  |
| 32 – 39; n,% | 104 | 0,1 | 0 | 0 |  |
| 40 – 47; n,% | 51 | 0,05 | 0 | 0 |  |
| 48 – 55; n,% | 27 | 0,03 | 0 | 0 |  |
| >56; n,% | 67 | 0,74 | * | 6,45 |  |
|  |  |  |  |  |  |
| **Complications** |  |  |  |  |  |
| Mechanical ventilation; n, % | 4.935 | 4,9 | 4 | 12,9 | 0,04 |
| Fluid and electrolyte disorders; n, % | 10.642 | 10,56 | 8 | 25,81 | 0,006 |
| Postpartum renal failure; n,% | 1.436 | 1,43 | 4 | 12,9 | <.0001 |
| Dialysis; n, % | 1.191 | 1,18 | 4 | 12,9 | <.0001 |
| Cardiopulmonary resuscitation; n,% | 687 | 0,68 | 3 | 9,68 | <.0001 |

Legend Supplemental Table 3: RBC, Red blood cell; Rh, rhesus

Note: * censored ≤3 patients
